# MRPL35 Induces Proliferation, Invasion, and Glutamine Metabolism in NSCLC Cells by Upregulating SLC7A5 Expression

**DOI:** 10.1111/crj.13799

**Published:** 2024-07-10

**Authors:** Wei Hou, Juan Chen, Yaoyuan Wang

**Affiliations:** ^1^ Respiratory Department Shaanxi Provincial Nuclear Industry 215 Hospital Xianyang China

**Keywords:** deubiquitination, glutamine metabolism, MRPL35, SLC7A5, USP39

## Abstract

**Background:**

Mitochondrial ribosomal protein L35 (MRPL35) has been reported to contribute to the growth of non–small cell lung cancer (NSCLC) cells. However, the functions and mechanisms of MRPL35 on glutamine metabolism in NSCLC remain unclear.

**Methods:**

The detection of mRNA and protein of MRPL35, ubiquitin‐specific protease 39 (USP39), and solute carrier family 7 member 5 (SLC7A5) was conducted using qRT‐PCR and western blotting. Cell proliferation, apoptosis, and invasion were evaluated using the MTT assay, EdU assay, flow cytometry, and transwell assay, respectively. Glutamine metabolism was analyzed by detecting glutamine consumption, α‐ketoglutarate level, and glutamate production. Cellular ubiquitination analyzed the deubiquitination effect of USP39 on MRPL35. An animal experiment was conducted for in vivo analysis.

**Results:**

MRPL35 was highly expressed in NSCLC tissues and cell lines, and high MRPL35 expression predicted poor outcome in NSCLC patients. In vitro analyses suggested that MRPL35 knockdown suppressed NSCLC cell proliferation, invasion, and glutamine metabolism. Moreover, MRPL35 silencing hindered tumor growth in vivo. Mechanistically, USP39 stabilized MRPL35 expression by deubiquitination and then promoted NSCLC cell proliferation, invasion, and glutamine metabolism. In addition, MRPL35 positively affected SLC7A5 expression in NSCLC cells in vitro and in vivo. Moreover, the anticancer effects of MRPL35 silencing could be rescued by SLC7A5 overexpression in NSCLC cells.

**Conclusion:**

MRPL35 expression was stabilized by USP39‐induced deubiquitination in NSCLC cells, and knockdown of MRPL35 suppressed NSCLC cell proliferation, invasion, and glutamine metabolism in vitro and impeded tumor growth in vivo by upregulating SLC7A5, providing a promising therapeutic target for NSCLC.

## Graphical abstract

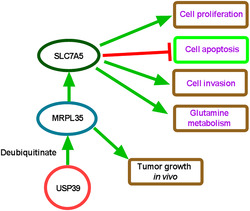

## 1 Introduction

Lung cancer is a leading cause of cancer death worldwide, with an estimated 135 000 deaths per year [1]. The most common histological type of lung cancer (≥ 80%) is non–small cell lung cancer (NSCLC), with a 5‐year survival rate of less than 20% [2]. Currently, the most effective therapy remains surgical resection, whereas the recurrence rate was 30% at 5 years after the surgery [3]. Moreover, the majority of NSCLC patients are diagnosed at the advanced stage owing to a lack of typical clinical manifestations and a loss of opportunity for surgery [4]. Thus, it is indispensable to develop novel and effective therapeutic strategies for NSCLC patients.

Currently, molecular targeted therapy adopting therapeutic monoclonal antibodies or small‐molecule agents that specifically suppress signal transduction involved in survival, growth, metabolism, and metastasis has been approved for the treatment of many cancer types [5, 6]. Mitochondria are the primary energy‐generating organelles, and mitochondrial function plays a pivotal role in tumorigenesis [7]. In addition to glycolysis, mitochondrial glutamine metabolism is the most common energy source for cancer cells. Glutamine is a nonessential amino acid (NEAA) that enters the cell via the amino acid transporters, such as ASCT2/SLC1A5, and is then catalyzed to glutamate by glutaminase in the mitochondria, and mitochondrial glutamate is subsequently converted into α‐ketoglutarate (α‐KG), which can help rapidly proliferating cancer cells meet the increasing demand for ATP and biosynthetic precursors and reducing agents [8, 9]. The survival and proliferation of proliferating cancer cells rely largely on glutamine metabolism [10]. In addition to their central role in modulating energy metabolism, mitochondria also serve as a fundamental backbone in the regulation of cell death, autophagy, apoptosis, control of redox and calcium homeostasis, and tumor anabolism [7, 11, 12]. The elimination of cancer cell mitochondrial DNA suppresses tumorigenicity [7]. Mitochondrial ribosomal proteins (MRPs) encoded by nuclear genes participate in the assemblage of mitoribosomes [13]. The alternation of mitochondrial ribosomal genes, including MRP genes, has been shown to be associated with cancer progression. For example, high MRPL35 expression was found in gastric cancer (GC), and the deficiency of MRPL35 suppressed GC growth [14]. MRPL35 silencing promoted ROS‐induced autophagy and apoptosis and suppressed growth in colorectal cancer [15]. Moreover, MRPL35 suppressed the growth of NSCLC by repressing proliferation‐ and apoptosis‐related genes [16]. However, the role and mechanism of MRPL35 on glutamine metabolism in NSCLC are still vague.

Herein, this study set out to probe the functions of MRPL35 in NSCLC cell growth, invasion, and glutamine metabolism and its possible molecular mechanism. This data first proposed the effects of the alternation of MRP genes on mitochondrial glutamine metabolism. The genetic inhibition of MRPs, such as MRPL35, may prevent NSCLC growth by blocking the dependence of cancer cells on glutamine metabolism, which may provide a novel insight into the development of molecular targeted therapy for NSCLC patients.

## 2 Materials and Methods

### 2.1 Clinical Samples

A total of 37 NSCLC patients diagnosed by pathological examinations were recruited. Written informed consent was obtained from every patient. Tumor tissues and paracarcinoma normal tissues (at least 5 cm distal to the tumor margin) were collected by surgery. The samples were preserved at −80 °C. The clinicopathologic features of NSCLC patients are shown in Table 1.

**TABLE 1:** Relationship between MRPL35 expression and clinicopathologic features of NSCLC patients.

	Characteristics (*n* = 37)	MRPL35 expression		*p* value
		Low (*n* = 18)	High (*n* = 19)	
Age (years)				0.7459
≤ 50	17	9	8	
> 50	20	9	11	
TNM grade				0.0217*
I + II	17	12	5	
III	20	6	14	
Lymph node metastasis				0.0009*
Positive	19	4	15	
Negative	18	14	4	
Tumor size				0.0025*
≤ 3 cm	21	15	6	
> 3 cm	16	3	13	

Abbreviation: TNM, tumor node metastasis.

* *p* < 0.05.

### 2.2 Cell Culture

Human NSCLC cell lines (Calu‐6, H1299, and A549) and human bronchial epithelial cell line 16HBE were purchased from Procell (Wuhan, China) and cultured in RPMI‐1640 (Procell) plus 10% fetal bovine serum (FBS, Procell) and 1% penicillin/streptomycin (Procell) with 5% CO_2_ at 37 °C.

### 2.3 Cell Transfection

The specific small interference RNAs (siRNAs) targeting USP39 or MRPL35 (si‐USP39: 5′‐TGGATTTGTACCATTCTTCTG‐3′ or si‐MRPL35: 5′‐CAACCTACCGCAACTGTGTCAAGAA‐3′) and the nontargeted siRNA (si‐NC: 5′‐CGGGAGGGTTATGTGCCAATAGCTA‐3′) were designed by GenePharma (Shanghai, China). The USP39 (NM_001256728.2), MRPL35 (NM_145644.3), or SLC7A5 (NM_003486.7) overexpression plasmids were contrasted by respectively cloning their full‐length into pcDNA3.1 plasmids (GenePharma), named USP39, MRPL35, or SLC7A5, and the empty plasmid was used as the control (pcDNA). Then, transient transfection was carried out using Lipofectamine 3000 (Invitrogen, Carlsbad, CA, USA).

### 2.4 Quantitative Real‐Time PCR (qRT‐PCR)

TRIzol reagent (Invitrogen) was adopted for extracting total RNAs, then the synthesis of cDNAs was conducted by reverse transcript using the PrimeScript™ RT kit (Takara, Dalian, China), followed by PCR amplification using the cDNA template, primers (Table 2), and the SYBR Green Taq Mix (Takara) according to the recommended protocol. The 2^‐∆∆Ct^ method was utilized for detecting gene levels with GAPDH as the normalization.

**TABLE 2:** The primers for qRT‐PCR.

Name		Primers for qRT‐PCR (5′‐3′)
MRPL35	Forward	TGCAAAGAAATTGGGTCTGTGT
	Reverse	GGTGACAACAGGGTGGTGAA
USP39	Forward	GAGTCTCGCGGTTCCACTC
	Reverse	CGGTCCTCAGAATCCACTCG
SLC7A5	Forward	GCTCATCATCCGGCCTTCAT
	Reverse	ATTGGACACATCACCCTTCCC
GAPDH	Forward	AGAAGGCTGGGGCTCATTTG
	Reverse	AGGGGCCATCCACAGTCTTC

### 2.5 Western Blotting

Total proteins were extracted using RIPA lysis buffer on ice (Yeasen, Shanghai, China). After determining the protein concentration, protein samples (about 40 μg per lane) were subjected to SDS‐PAGE for separation and then shifted onto the PVDF membranes (Beyotime, Beijing, China). Next, the antibodies against USP39 (#PA5‐97229, 1:1000), MRPL35 (#PA5‐101361, 1:1000), SLC7A5 (#PA5‐115916, 1:2000), and GAPDH (#MA5‐15738, 1:2000) (Invitrogen) were used to incubate with PVDF membranes for 12 h at 4 °C. After secondary incubation with HRP‐linked antibodies, protein signals were determined using the ECL substrate kit (Amersham, Piscataway, NJ).

### 2.6 MTT Assay

After indicated transfection, H1299 and A549 cells (1 × 10^4^ cells/well) were incubated for 4 h with 20 μL MTT (5 mg/mL) (Beyotime) in a 96‐well plate. Then, 200 μL DMSO was added into each well to dissolve MTT formazan crystals, and the absorbance was tested at 570 and 630 nm.

### 2.7 5‐Ethynyl‐2‐Deoxyuridine (EdU) Assay

Following the indicated transfection, H1299 and A549 cells were reacted with 50 μM EdU solution (RiboBio, Guangdong, China) in a 96‐well plate for 3 h. Then, cells were fixed with 4% paraformaldehyde (Beyotime) for 0.5 h and permeabilized using Triton‐X‐100 (0.5%), followed by dyeing with DAPI (Invitrogen) for 10 min. Lastly, EdU‐positive cells were captured and counted.

### 2.8 Flow Cytometry

Assigned H1299 and A549 cells were resuspended in 1 × binding buffer and then labeled with 10 μL Annexin V‐FITC and 10 μL propidium iodide (PI) (KeyGen, Nanjing, China), avoiding light for 15 min. Finally, apoptotic cells were analyzed using a flow cytometer.

### 2.9 Transwell Assay

The filters of the transwell chamber (Corning Costar, Corning, NY, USA) were precoated with 100 μL of Matrigel (1 mg/mL). Then, the assigned H1299 and A549 cells resuspended in serum‐free RPMI‐1640 were added into the upper chambers, and 500 μL of medium containing 10% FBS was filled into the lower chamber. Twenty‐four hours later, invaded cells were fixed with 4% paraformaldehyde (Beyotime) and dyed with 0.1% crystal violet (Beyotime). Then, cells were observed and counted.

### 2.10 Glutamine Metabolism Analysis

The supernatants of assigned H1299 and A549 cells were collected, and glutamine consumption was determined as per the recommended instructions of a glutamine assay kit (Colorimetric) (Abcam, Cambridge, UK).

H1299 and A549 cells following indicated transfection were homogenized in PBS, then supernatants were harvested and levels of α‐KG and glutamate were measured as per the protocol of commercial kits (Abcam) after removing the proteins.

### 2.11 Ubiquitination Assay

The expression vectors Flag‐tagged MRPL35 were transfected into 293 T cells that were pretransfected with si‐NC or si‐USP39. MRPL35 was first immunoprecipitated by anti‐Flag antibody and protein A/G magnetic beads (Thermo Fisher Scientific, Inc., Waltham, MA, USA). Then, cells were lysed and incubated with a ubiquitin (Ub) antibody (ab134953, Abcam) to detect the ubiquitination of MRPL35 by western blotting.

### 2.12 Animal Experiments

The sh‐NC or sh‐MRPL35 (GenePharma) was cloned into the lentiviral plasmids (ATCC, Rockville, MD, USA) and then transfected into 293 T cells. Forty‐eight hours later, the supernatants of 293 T cells were collected and ultracentrifuged at 72 000 × g for 2 h. Then, lentiviral particles carrying sh‐NC or sh‐MRPL35 were collected and incubated for 12 h with A549 cells in a completed medium containing 8 μg/mL polybrene. The BALB/c nude mice (4–5 weeks old, male) were purchased from Slaike Jingda Laboratory (Hunan, China) and subcutaneously injected with infected A549 cells. Then, mice were divided into four groups (*n* = 6 per group): sh‐NC, sh‐MRPL35, sh‐MRPL35 + pcDNA, or sh‐MRPL35 + SLC7A5. Eight days after inoculation, mice in the sh‐MRPL35 + pcDNA or sh‐MRPL35 + SLC7A5 group were treated with pcDNA or SLC7A5 in Lipofectamine through a local injection at 4–5 sites of the xenograft tumor. Tumor volume was recorded every 3 days and calculated by the following formula: volume = 0.5 × width^2^ × length. At the end of time, mice were killed, and xenograft tumors were isolated and weighed.

### 2.13 Immunohistochemistry (IHC) Analysis

The paraffin‐embedded human NSCLC tumor and normal tissues or mouse xenograft tumors were prepared and then cut into 4 μm tissue sections. The sections were subjected to deparaffinization, rehydration, and antigen retrieval. Then, sections were labeled with the primary antibodies overnight at 4 °C and secondary antibodies for 2 h at 37 °C, followed by staining with diaminobenzidine (DAB) (Beyotime).

### 2.14 Statistical Analysis

The data were manifested as mean ± standard deviation (SD). Statistical analyses were carried out using Prism 8 (GraphPad Software, San Diego, CA, USA). The differences were analyzed using Student's *t*‐test (two groups) or ANOVA (multiple groups). Correlation analysis was conducted using the Pearson correlation test. *p* < 0.05 means statistically significant.

## 3 Results

### 3.1 MRPL35 Is Highly Expressed in NSCLC Tissues and Cell Lines

**FIGURE 1:**
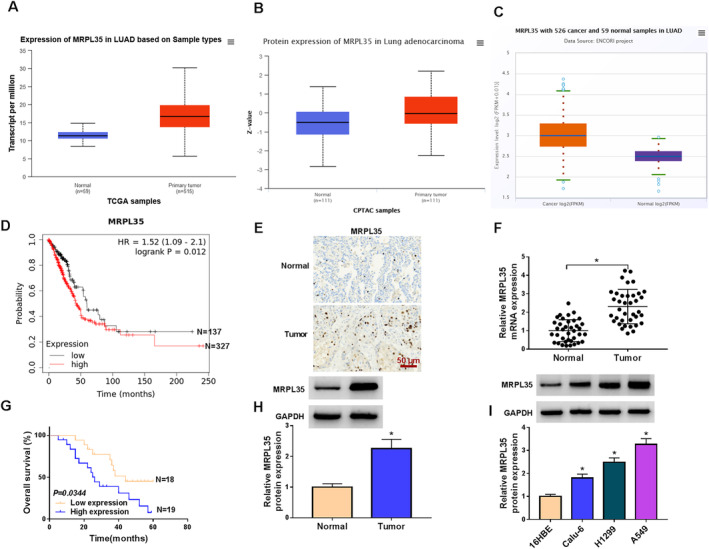
MRPL35 is highly expressed in NSCLC tissues and cell lines. (A–C) TCGA, CPTAC, and ENCORI databases showed the high expression of MRPL35 in lung cancer tissues. (D) The Kaplan–Meier plotter database suggested that high MRPL35 expression was associated with shorter survival times in NSCLC patients. (E) IHC analysis for MRPL35 expression in NSCLC tissues and normal tissues. (F) qRT‐PCR analysis for MRPL35 mRNA expression in NSCLC tissues and normal tissues. (G) Kaplan–Meier overall survival curves for 37 NSCLC patients classified according to relative MRPL35 expression level. (H, I) Western blotting analysis for MRPL35 protein expression in NSCLC tissues and normal tissues, as well as in NSCLC cell lines and normal 16HBE cells. *p < 0.05.

According to the TCGA, CPTAC, and ENCORI databases, we found that MRPL35 levels were higher in lung cancer tissues than in normal tissues (Figure 1A–C). Moreover, the Kaplan–Meier plotter database showed that high MRPL35 expression was associated with shorter survival times (Figure 1D). Then, the expression profiles of MRPL35 in clinical samples were investigated. IHC staining suggested a high positive for MRPL35 in NSCLC tissues (Figure 1E). Further, qRT‐PCR analysis exhibited a higher expression of MRPL35 mRNA in NSCLC tissues (Figure 1F). Moreover, high MRPL35 expression predicted a poor survival rate in NSCLC patients (Figure 1G). Also, MRPL35 protein levels were increased in NSCLC tissues relative to normal tissues (Figure 1H). Additionally, higher MRPL35 expression was correlated with advanced TNM stages, lymph node metastasis, and big tumor size (Table 1). In addition, NSCLC cell lines also showed highly expressed MRPL35 compared with the 16HBE cells (Figure 1I).

### 3.2 MRPL35 Knockdown Suppresses NSCLC Cell Proliferation, Invasion, and Glutamine Metabolism and Induces Cell Apoptosis

**FIGURE 2:**
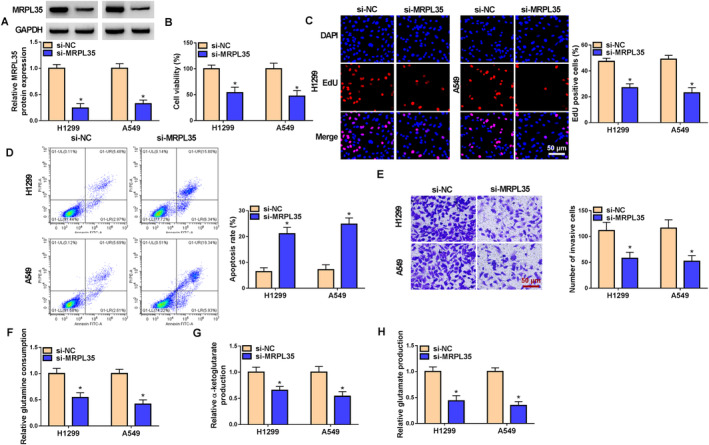
MRPL35 knockdown suppresses NSCLC cell proliferation, invasion, and glutamine metabolism and induces cell apoptosis. (A–H) H1299 and A549 cells were transfected with si‐MRPL35 or si‐NC. (A) Western blotting analysis for MRPL35 protein expression in cells. (B, C) Measurement of cell proliferation by MTT and EdU assays. (D) Flow cytometry for cell apoptosis. (E) Transwell assay for cell invasion analysis. (F–H) Glutamine metabolism analysis by detecting glutamine consumption and the production of α‐ketoglutarate and glutamate. *p < 0.05.

Next, the functions of MRPL35 in NSCLC cells were studied. Western blotting analysis showed that si‐MRPL35 introduction markedly reduced the expression level of MRPL35 in H1299 and A549 cells (Figure 2A). Functionally, MRPL35 silencing suppressed the viability and DNA synthesis activity in H1299 and A549 cells, suggesting the inhibition of cell proliferation (Figure 2B,C). On the contrary, MRPL35 silencing induced apoptosis in H1299 and A549 cells (Figure 2D). Moreover, the number of invaded H1299 and A549 cells was also reduced after MRPL35 silencing (Figure 2E). In addition, MRPL35 deficiency suppressed glutamine metabolism in H1299 and A549 cells, demonstrated by decreased glutamine consumption and the production of α‐KG and glutamate (Figure 2F–H).

### 3.3 USP39 Induces MRPL35 Deubiquitination and Stabilizes Its Expression

**FIGURE 3:**
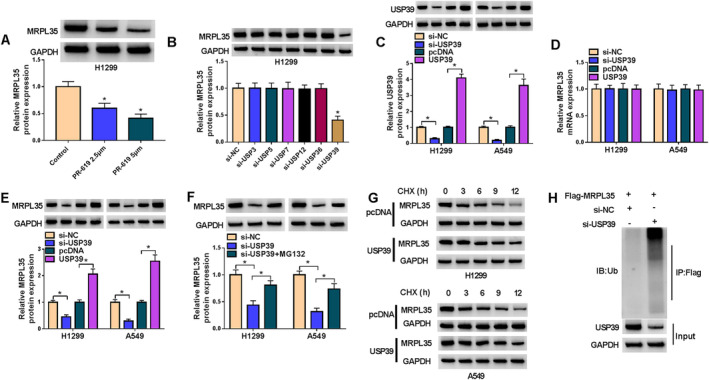
USP39 induces MRPL35 deubiquitination and stabilizes its expression. (A) Western blotting analysis for MRPL35 protein expression in H1299 and A549 cells after incubating with PR‐619 (2.5 or 5 μm). (B) The effects of USP enzyme families on MRPL35 protein expression in H1299 and A549 cells were investigated by western blotting. (C) The transfection efficiencies of si‐USP39, USP39, or the control (si‐NC or pcDNA) were verified by detecting USP39 levels by western blotting in NSCLC cells. (D, E) qRT‐PCR and western blotting analyses for MRPL35 protein expression in NSCLC cells after USP39 silencing or overexpression. (F) NSCLC cells were transfected with si‐NC, si‐USP39, or si‐USP39 + MG132, and MRPL35 protein expression was measured by western blotting. (G) Western blotting for MRPL35 protein in NSCLC cells after USP39 overexpression under CHX treatment. (H) IP and western blotting assays detected ubiquitin‐MRPL35 levels in 293 T cells with indicated antibodies. *p < 0.05.

As shown in Figure 3A, we found that the incubation of PR‐619, a deubiquitinating enzyme (DUB) inhibitor, led to a decrease in MRPL35 levels in H1299 cells, indicating that MRPL35 may be stabilized by deubiquitination through DUBs. Then, we found that only the knockdown of USP39 reduced MRPL35 expression in H1299 cells compared with other USP enzyme families (Figure 3B). After confirming the transfection efficiencies of si‐USP39 or USP39 (Figure 3C), we found that USP39 silencing or overexpression did not affect the expression of MRPL35 mRNA in NSCLC cells (Figure 3D). However, MRPL35 protein expression was reduced by USP39 silencing but increased by USP39 overexpression in H1299 and A549 cells (Figure 3E). In addition, the proteasome inhibitor MG‐132 (a protein degradation inhibitor) could suppress the si‐USP39‐induced decrease of MRPL35 protein in H1299 and A549 cells (Figure 3F), implying that the MRPL35 downregulation was caused in a Ub‐proteasome‐dependent manner. Besides that, H1299 and A549 cells were incubated with cycloheximide (CHX), a chemical that inhibits protein synthesis, for different times, and then western blotting analysis showed that USP39 overexpression stabilized MRPL35 protein expression in cells (Figure 3G). Moreover, we transfected Flag‐MRPL35 and si‐NC or si‐USP39 into 293 T cells and found that USP39 silencing enhanced MRPL35 ubiquitination (Figure 3H).

### 3.4 USP39 Knockdown Suppresses NSCLC Cell Proliferation, Invasion, and Glutamine Metabolism and Induces Cell Apoptosis by MRPL35

**FIGURE 4:**
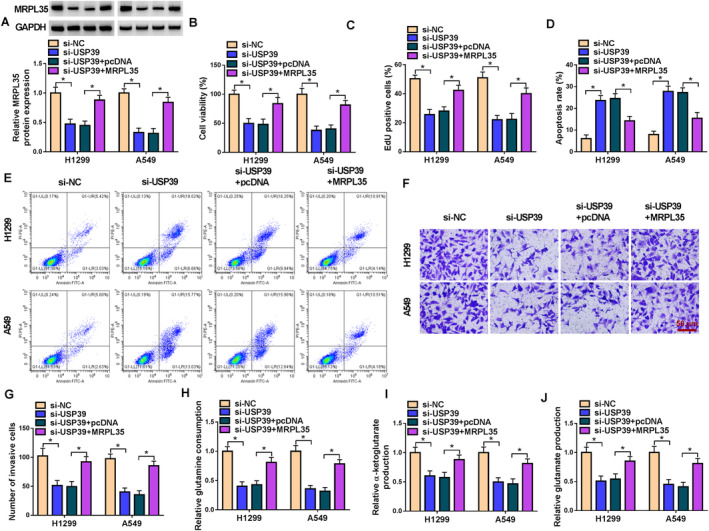
USP39 knockdown suppresses NSCLC cell proliferation, invasion, and glutamine metabolism and induces cell apoptosis by MRPL35. (A–I) H1299 and A549 cells were transfected with si‐USP39 or si‐USP39 and MRPL35. (A) Western blotting analysis for MRPL35 protein expression in cells. (B, C) Measurement of cell proliferation by MTT and EdU assays. (D, E) Flow cytometry for cell apoptosis. (F, G) Transwell assay for cell invasion analysis. (H–J) Glutamine metabolism analysis by detecting glutamine consumption and the production of α‐ketoglutarate and glutamate. *p < 0.05.

Subsequently, we investigated whether the effects of MRPL35 on NSCLC cells were mediated by USP39. H1299 and A549 cells were transfected with si‐USP39 or si‐USP39 and MRPL35. Figure 4A showed that MRPL35 introduction rescued a si‐USP39‐induced decrease of MRPL35 in NSCLC cells. Functionally, si‐USP39 suppressed the proliferation (Figure 4B,C), triggered the apoptosis (Figure 4D,E), and impaired the invasion (Figure 4F,G) in H1299 and A549 cells. Moreover, USP39 deficiency suppressed glutamine metabolism, while this effect was reversed by MRPL35 overexpression, evidenced by increased glutamine consumption and the production of α‐KG and glutamate (Figure 4H–J).

### 3.5 SLC7A5 Expression Is Higher in NSCLC Tissues and Cells and Is Affected by MRPL35

**FIGURE 5:**
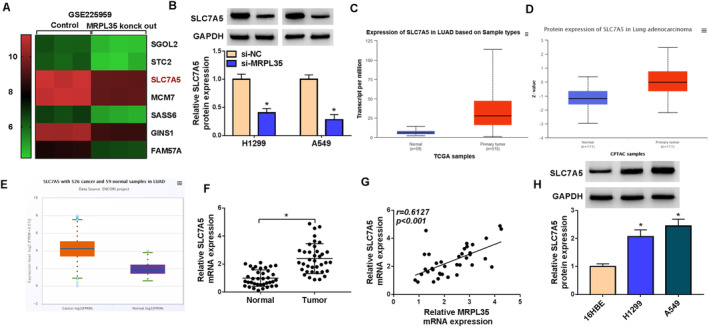
SLC7A5 expression is higher in NSCLC tissues and cells and is affected by MRPL35. (A) Heat map of deregulated genes in A549 cells after MRPL35 knockdown according to the GSE225959 dataset. (B) Protein levels of SLC7A5 in H1299 and A549 cells after MRPL35 knockdown. (C–E) TCGA, CPTAC, and ENCORI databases showed the high expression of SLC7A5 in lung cancer tissues. (F) qRT‐PCR analysis for SLC7A5 in NSCLC tissues and normal tissues. (G) Correlation analysis between SLC7A5 and MRPL35 mRNA in NSCLC tissues. (H) Western blotting analysis for SLC7A5 protein expression in NSCLC cell lines and normal 16HBE cells. *p < 0.05.

According to the GSE225959 dataset, the heat map of deregulated genes in A549 cells after MRPL35 knockdown is shown in Figure 5A. We found that SLC7A5, an amino acid transporter associated with glutamine consumption, was decreased in A549 cells after MRPL35 knockdown compared with the control (Figure 5A). Then, we also demonstrated that MRPL35 silencing reduced the expression of SLC7A5 in H1299 and A549 cells (Figure 5B). Thereafter, the TCGA, CPTAC, and ENCORI databases exhibit the high expression of SLC7A5 in lung cancer tissues (Figure 5C–E). In clinical NSCLC samples, we also observed an increased expression of SLC7A5 relative to the normal tissues (Figure 5F). And SLC7A5 mRNA expression was positively correlated with MRPL35 expression in NSCLC tissues (Figure 5G). Also, SLC7A5 expression was higher in NSCLC cell lines (Figure 5H).

### 3.6 MRPL35 Knockdown Suppresses NSCLC Cell Proliferation, Invasion, and Glutamine Metabolism and Induces Cell Apoptosis by Regulating SLC7A5

**FIGURE 6:**
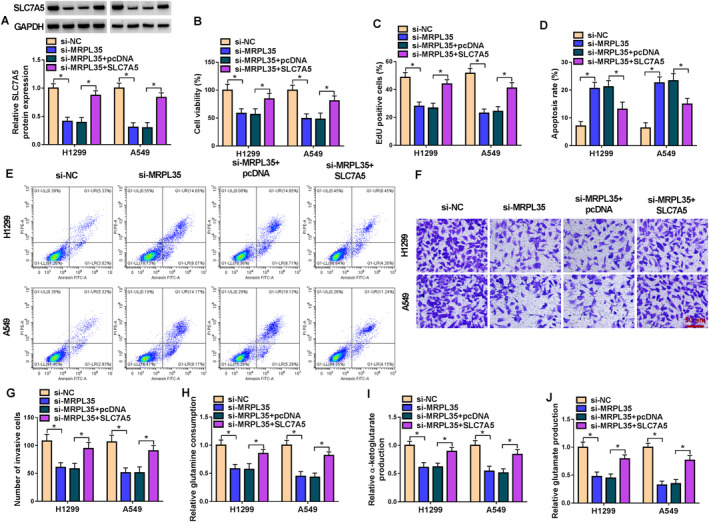
MRPL35 knockdown suppresses NSCLC cell proliferation, invasion, and glutamine metabolism and induces cell apoptosis by regulating SLC7A5. (A–I) H1299 and A549 cells were transfected with si‐MRPL35 or si‐MRPL35 and SLC7A5. (A) Western blotting analysis for SLC7A5 protein expression in cells. (B, C) Measurement of cell proliferation by MTT and EdU assays. (D, E) Flow cytometry for cell apoptosis. (F, G) Transwell assay for cell invasion analysis. (H–J) Glutamine metabolism analysis by detecting glutamine consumption and the production of α‐ketoglutarate and glutamate. *p < 0.05.

Thereafter, we studied whether MRPL35 modulated NSCLC cells by affecting SLC7A5 expression. SLC7A5 overexpression plasmids were established. Then, Figure 6A showed that SLC7A5 introduction rescued MRPL35 knockdown‐induced SLC7A5 decrease in H1299 and A549 cells (Figure 6A). Further functional analyses indicated that SLC7A5 overexpression abolished MRPL35 silencing‐induced proliferation inhibition (Figure 6B,C), apoptosis enhancement (Figure 6D,E), and invasion arrest (Figure 6F,G) in H1299 and A549 cells. Besides that, the decreases in glutamine consumption, α‐KG production, and glutamate contents caused by MRPL35 silencing were reverted by SLC7A5 upregulation (Figure 6H–J).

### 3.7 MRPL35 Knockdown Impedes NSCLC Tumor Growth In Vivo

**FIGURE 7:**
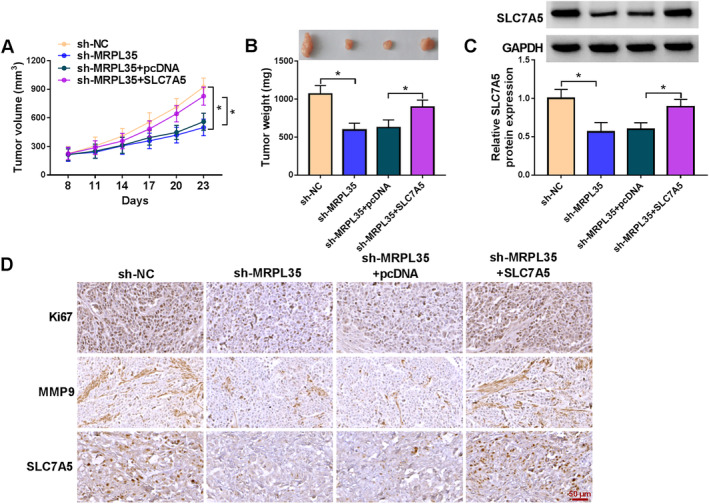
MRPL35 knockdown impedes NSCLC tumor growth in vivo. (A) Tumor volume of mice in each group. (B) Representative xenografts and tumor weight in each group. (C) Western blotting analysis for SLC7A5 protein expression in xenografts of each group. (D) IHC staining for Ki67, MMP9, and SLC7A5 protein in xenografts of each group. *p < 0.05.

Finally, we explored the role of MRPL35 in NSCLC growth in vivo. As exhibited in Figure 7A,B, we found that MRPL35 silencing reduced the volume and weight of xenografts, which was rescued by SLC7A5 upregulation. Moreover, SLC7A5 levels were decreased in xenografts of the sh‐MRPL35 group but increased in xenografts of the sh‐MRPL35 + SLC7A5 group (Figure 7C). In addition, IHC staining suggested that the positive cells of Ki67, MMP9, and SLC7A5 in xenografts of the sh‐MRPL35 group were reduced, which were rescued in xenografts of the sh‐MRPL35 + SLC7A5 group (Figure 7D), indicating that MRPL35 might suppress NSCLC cell proliferation and invasion by regulating SLC7A5 in vivo.

## 4 Discussion

To date, the dysfunction of mitochondria has been demonstrated to contribute to cancer progression by different mechanisms [17]. MRPs encoded by nuclear genes are involved in the assembly of mitoribosomes and help in the translation of mitochondrial proteins [18]. High MRPL13 expression was related to advanced stage and poor survival in NSCLC patients, and the silencing of MRPL13 suppressed cancer cell proliferation [19]. Highly expressed MRPL15 predicted poor outcomes in NSCLC; might be involved in modulating DNA replication, metabolism‐associated pathways, and cell cycle signaling; and was negatively related to immune infiltration [20]. In addition, MRPL42 deficiency suppressed proliferation, invasion and migration, arrested the cell cycle in lung adenocarcinoma cells in vitro, and hampered tumor growth in vivo [21]. Thus, the deregulation of MRP genes plays an important role in lung cancer progression. In our study, a high expression of MRPL35 in NSCLC patients was observed, and MRPL35 indicated poor prognosis in patients. Functionally, MRPL35 deficiency repressed NSCLC growth in vitro and in vivo, which was consistent with previous findings [16]. Moreover, we also found that MRPL35 silencing impaired cell invasion. Cancer is hallmarked by the deregulation of cellular energetic metabolism. Herein, we observed decreased glutamine consumption, α‐KG production, and glutamate generation after MRPL35 silencing in NSCLC cells, implying that MRPL35 silencing induced glutamine metabolism inhibition to prevent cancer cells from gaining energy. Thus, MRPL35 siRNAs or shRNAs may be promising molecules for NSCLC‐targeted therapy.

Next, we investigated the mechanism of MRPL35 on NSCLC progression. We proved that USP39 induced MRPL35 deubiquitination to stabilize its expression. USP39 is a member of USPs, the largest DUB [22]. The removal of Ub, Ub‐like molecules, or remodeling of Ub from protein substrates mediated by DUBs is termed deubiquitination, which can preserve substrate protein expression, thereby regulating physiological pathways [23]. USP39 has been identified as participating in tumorigenesis. For instance, USP39 silencing enhanced cisplatin‐induced apoptosis in colon cancer cells by increasing p53 expression [24]. USP39 deficiency activates p53 pathway–induced apoptosis and metastasis in NSCLC [25]. In the present study, we observed that USP39 deletion could suppress NSCLC cell proliferation, invasion, and glutamine metabolism and induce cell apoptosis, while these effects were reversed after MRPL35 overexpression, indicating that the effects of MRPL35 on NSCLC might be associated with USP39‐induced deubiquitination. In addition, we also found that MRPL35 positively affected the expression of SLC7A5. SLC7A5, also known as LAT1, is a glutamine antiporter. It is critical for the efficient uptake of leucine and glutamine and then sends a regulatory signal to activate the mammalian target of rapamycin (mTOR), thereby supporting cancer cell proliferation, growth, and apoptosis resistance [26, 27]. Interestingly, SLC7A5 has been identified as being upregulated in many cancers and closely linked with the growth and proliferation of cancer cells [28, 29]. In lung cancer, Liu et al. showed that SLC7A5 silencing could reduce tumorsphere formation and cancer stemness by impairing the activation of mTOR and reducing PD‐L1 expression [30]. The downregulation of glutamine transporters (ASCT2 and LAT1) was able to reduce the uptake of glutamine in proliferating cells, thereby repressing NSCLC cell proliferation and inducing cell apoptosis by blocking the mTOR pathway [31]. In this work, we confirmed that SLC7A5 expression was higher in NSCLC and was positively correlated with MRPL35 in NSCLC tissues. Moreover, SLC7A5 overexpression reversed MRPL35 knockdown‐caused inhibition of NSCLC cell growth, invasion, glutamine metabolism, and induction of cell apoptosis, implying that MRPL35 modulated NSCLC progression by SLC7A5.

In conclusion, this study demonstrated that MRPL35 contributed to NSCLC cell growth, invasion, and glutamine metabolism by upregulating SLC7A5 expression, and its mechanism might also be related to USP39‐mediated MRPL35 deubiquitination. Currently, targeting cancer cell–dependent glutamine metabolism has attracted great interest for anticancer therapeutics. Many classes of compounds that target glutamine metabolism, from initial intracellular transport to conversion to α‐KG, have been analyzed; moreover, one of the allosteric inhibitors of glutaminase, CB‐839, has moved on to clinical trials [32]. Thus, these data suggest a new insight into the development of molecular targeted therapy based on glutamine metabolism for NSCLC patients.

## Author Contributions

Wei Hou conducted the experiments and drafted the manuscript. Juan Chen collected and analyzed the data and prepared the figures. Yaoyuan Wang designed and supervised the study. All authors reviewed the manuscript.

## Ethics Statement

This study was performed by the institutional ethics committee of Shaanxi Provincial Nuclear Industry 215 Hospital based on the Declaration of Helsinki.

This animal study was approved by the Ethics Committee of the Shaanxi Provincial Nuclear Industry 215 Hospital.

## Conflicts of Interest

The authors declare no conflicts of interest.

## Data Availability

The data that support the findings of this study are available from the corresponding author upon reasonable request.
